# Rate-based structural health monitoring using permanently installed sensors

**DOI:** 10.1098/rspa.2017.0270

**Published:** 2017-09-13

**Authors:** Joseph Corcoran

**Affiliations:** Mechanical Engineering Department, Imperial College London, London, UK

**Keywords:** structural health monitoring, permanently installed sensors, monitoring, remnant life, non-destructive evaluation, positive feedback

## Abstract

Permanently installed sensors are becoming increasingly ubiquitous, facilitating very frequent *in situ* measurements and consequently improved monitoring of ‘trends’ in the observed system behaviour. It is proposed that this newly available data may be used to provide prior warning and forecasting of critical events, particularly system failure. Numerous damage mechanisms are examples of positive feedback; they are ‘self-accelerating’ with an increasing rate of damage towards failure. The positive feedback leads to a common time-response behaviour which may be described by an empirical relation allowing prediction of the time to criticality. This study focuses on Structural Health Monitoring of engineering components; failure times are projected well in advance of failure for fatigue, creep crack growth and volumetric creep damage experiments. The proposed methodology provides a widely applicable framework for using newly available near-continuous data from permanently installed sensors to predict time until failure in a range of application areas including engineering, geophysics and medicine.

## 1. Introduction

Advances in battery power, wireless communication and transducers make permanently installed sensors an increasingly attractive proposition; wearable sensors [1,2], ‘connected devices’ [3,4] and structural health monitoring (SHM) systems [5,6] are becoming increasingly common. Permanently installed sensors facilitate frequent measurements and therefore introduce improved time domain resolution to situations where previously manual inspection-type measurements were infrequent. A key motivation for the frequent data collection is to establish ‘trends’ in data; it is proposed in this study that monitoring the rate of change of damage, or symptoms of damage, will provide warning to preclude critical events, in particular system failure. This paper concentrates on the use of permanently installed sensors for the structural health monitoring of engineering components, but it is emphasized that the key points are more generally applicable beyond engineering.

‘Damage tolerant’ engineering components are designed so that they are able to sustain defects and safely operate until appropriate measures are decided and enacted [5]. Equally, many components are designed with the acceptance that they will gradually degrade through normal operation; high temperature components frequently have a finite usable life due to thermally activated damage mechanisms [7–9]. In both cases it is necessary to assess the expected remnant life of the components. The failure time is specific to the particular combination of macroscopic and microscopic integrity coupled with the operating conditions and environmental influences. Conventionally, in engineering applications, the risk associated with the presence of damage is managed through non-destructive evaluation (NDE) inspections combined with fracture mechanics based structural integrity assessment techniques [10–12]. Such an approach is prone to significant errors resulting from the need to estimate the present damage, material properties and stress state, which are not trivial to measure directly in an industrial context. This may be seen as analogous to a human with a progressive illness, or whose health is declining with age; again, the deterioration of the system will be specific to a wide range of parameters which may not be well characterized and individual measurements of damage severity may be infrequent and difficult to obtain. It is proposed that in both cases the introduction of frequent measurements of symptoms of damage will provide the situation specific rate of decline of the system which is necessary to forecast failure.

**Figure 1.**
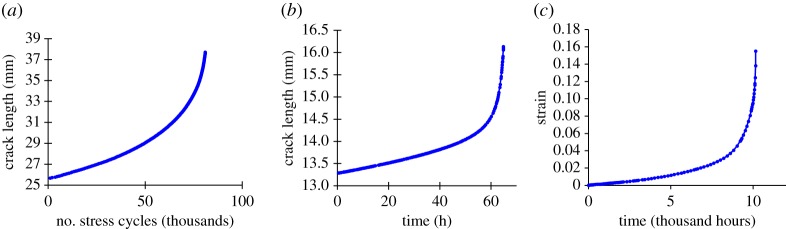
Example experimental data showing similar increasing rate with proximity to failure behaviour for three damage mechanisms; the specific details of each experiment are given later in this paper, they are shown here to illustrate general behaviour. (*a*) Fatigue, a constant amplitude load cycle is applied to a pre-cracked component at room temperature. Data from [13]. (*b*) Creep crack growth, a constant load is applied to a pre-cracked test component at 600°C. Data from [14]. (*c*) Volumetric creep, a constant load is applied to a cylindrical uniaxial creep test component at 650°C. Data from [15]. (Online version in colour.)

In many cases system failures are a consequence of positive feedback. Damage compromises the capacity of the system to sustain ‘load’, which in turn leads to an increased rate of damage accumulation; the deterioration of the system ‘self-accelerates’ until failure. Positive feedback mechanisms are frequently unstable and are characterized by an increasing rate of change approaching failure, as shown in figure 1 for three examples of material failure mechanisms. Clearly, the increase in rate is indicative of proximity to failure and could potentially be interpreted for remnant life estimates. An empirical relation, originally proposed by Voight [16,17], is adopted which provides a rate-based framework for predicting failure time known as the ‘Failure Forecast Method’.

The use of rate-based assessment is given renewed relevance with the increasing feasibility of near-continuous monitoring using permanently installed sensors. The confidence interval associated with a rate estimate is proportional to the square root of the time interval between successive measurements, as demonstrated in the analysis in the electronic supplementary material, §S1; reducing the interval between measurements is therefore critical for satisfactory rate calculations. Conventionally, damage is characterized at convenient opportunities using manual NDE inspection techniques. In many cases, particularly where the component operates at high temperature or is difficult to access, then the inspections may be separated by long periods, frequently years, and therefore the resulting rate calculations are unlikely to be satisfactory. Permanently installed sensors enable frequent measurements, where the time between measurements is often less than a day (www.permasense.com (2015). See http://www.permasense.com/), leading to improved rate calculations. Additionally, permanently installed sensors help suppress measurement variation attributed to equipment, operator and location uncertainty. The term ‘damage’ has been left deliberately vague in this study. As it is not strictly the damage that is being interpreted but *an increase in the rate of damage,* far more general metrics can be used. Metrics which are symptomatic of the underlying positive feedback mechanism may conform to the same behaviour and are therefore interpretable in the same way.

A coupled approach is proposed combining the empirical behaviour of positive feedback mechanisms with the use of frequent measurements from permanently installed sensors to preclude a point of criticality well in advance of failure. The following section will provide background to positive feedback damage mechanisms and review the Failure Forecast Method. Following this, fatigue, volumetric creep and creep crack growth will be used as illustrative examples of positive feedback mechanisms; for each mechanism conformity to Voight's proposed behaviour and therefore compatibility with the Failure Forecast Method will be shown. For each mechanism experimental data will be used to demonstrate the use of the methodology for remnant life prediction. Finally, discussion on the successful implementation of the methodology will be given followed by conclusions.

## 2. Positive feedback damage mechanisms and the Failure Forecast Method

Feedback mechanisms are described by the general relationship,

2.1 $$\frac{\text{d}\Omega }{\text{d}t}=f(\Omega ).$$

The feedback is positive when an increase in the quantity, *Ω*, leads to an increase in the rate of change of the quantity. Many load-controlled material damage mechanisms are examples of positive feedback mechanisms. Throughout this study, fatigue, creep crack growth and volumetric creep are used as illustrative examples; in fatigue and creep crack growth ‘damage’ is considered as crack length, while in volumetric creep it is strain. This study seeks to provide a means of projecting the time of the failure asymptote based on behaviour earlier on in the component life.

Following from the work of Fukuzono [18], Voight [16,17] observed ‘striking generality’ in a range of ‘rate-dependent’ failure mechanisms. He proposed a simple empirical relationship describing the behaviour of a wide range of systems;

2.2 $$\frac{{\text{d}}^{2}\Omega }{\text{d}t^{2}}=A{\left(\frac{\text{d}\Omega }{\text{d}t}\right)}^{\alpha },$$

where *α* and *A* are ‘constants of experience’ for the situation at hand, and additionally observed that frequently *α ≈ *2. The relationship provides the foundation for a framework, later termed the ‘Failure Forecast Method’, relating the increase in the rate of damage metrics to the proximity of the asymptotic criticality. Voight's work was developed in geophysics applications where continuous monitoring has been common place for some time [19]; an increase in the rate of seismic activity or deformation is related to the proximity to critical events such as volcanic eruptions [16,19–25] and landslides [21,26,27].

This study proposes that the cause of the ‘striking generality’ is positive feedback. The form of the function in equation (2.1) controls the behaviour of the component, relating the present damage state to rate of change of damage, which ultimately dictates the time-response of the component. As *Ω* is a function of time, equation (2.1) is differentiated

2.3 $$\frac{{\text{d}}^{2}\Omega }{\text{d}t^{2}}=\frac{\text{d}f(\Omega )}{\text{d}\Omega }\frac{\text{d}\Omega }{\text{d}t}.$$

The basis for Voight's postulation is now evident. If the positive feedback mechanism conforms to the criterion

2.4 $$\frac{\text{d}f(\Omega )}{\text{d}\Omega }=A{\left(\frac{\text{d}\Omega }{\text{d}t}\right)}^{\alpha -1},$$

then it can be combined with equations (2.3) to arrive at equation (2.2),

2.5 $$\frac{{\text{d}}^{2}\Omega }{\text{d}t^{2}}=A{\left(\frac{\text{d}\Omega }{\text{d}t}\right)}^{\alpha -1}\frac{\text{d}\Omega }{\text{d}t}=A{\left(\frac{\text{d}\Omega }{\text{d}t}\right)}^{\alpha }.$$

Therefore, the criterion for a positive feedback mechanism to conform to Voight's postulated relationship may be summarized as:

2.6 $$\frac{\text{d}f(\Omega )}{\text{d}\Omega }=Af{\left(\Omega \right)}^{\alpha -1},$$

i.e. if the derivative of the positive feedback function is a power relationship of itself, then the mechanism will adhere to Voight's widely observed behaviour. This forms a condition that can be used to check for agreement with Voight's postulation. A very wide range of functions fulfil this condition. Examples are given in table 1 with their derivatives.

**Table 1.** Examples of forms of positive feedback mechanisms, together with their derivatives, illustrating conformity with the form of equation (2.6).

dΩ Adt j=f(Ω)=kΩn	(2.7*a*)	df(Ω) AdΩ=nkΩn−1=nk1/n f(Ω)(n−1)/n≡Af(Ω)α−1	(2.7*b*)
dΩ Adt j=f(Ω)=kabΩ	(2.8*a*)	df(Ω) AdΩ=b ln(a) k abΩ=b ln(a) f(Ω)≡Af(Ω)α−1	(2.8*b*)
*including the case where a is Euler's constant e≈2.718*			
$\frac{\text{d}\Omega }{\text{d}t}=f(\Omega )=ke^{b\Omega }$	(2.9*a*)	df(Ω) AdΩ=bkebΩ=bf(Ω)≡Af(Ω)α−1	(2.9*b*)

The observation that *α ≈ *2 is given further foundation with equation (2.6). If the exponent in equation (2.6), (*α* − 1), is approximately 1 then *α* must clearly be approximately 2. This is seen to be exactly the case for equations (2.6) and (2.7), and, in equation (2.5) if *n* > 1 *α* is bound between 1 and 2 and as *n* becomes large *α* tends to 2.

Voight's postulation is significant as it can be integrated to give remnant life estimates. For *α* *>* 1, equation (2.2) can be integrated to give [17],

2.10 $$\frac{\text{d}\Omega }{\text{d}t}={\left[A(\alpha -1)(t_{f}-t)+{\left(\frac{\text{d}\Omega }{\text{d}t}\right)}_{f}^{1-\alpha }\right]}^{1/1-\alpha },$$

where the subscript f denotes the value at failure time. As the rate at failure is observed to be much larger than the rate earlier on in the component life then it will be assumed that the inverse failure rate is negligible allowing the simplification,

2.11 $$t_{f}-t=\frac{1}{A(\alpha -1)}{\left(\frac{1}{\text{d}\Omega \text{/d}t}\right)}^{\alpha -1}.$$

There is therefore a simple general relation between inverse rate and remnant life for a wide variety of positive feedback mechanisms. Equation (2.11) is further simplified if *α ≈ *2 leading to inverse proportionality between rate and remnant life.

**Figure 2.**
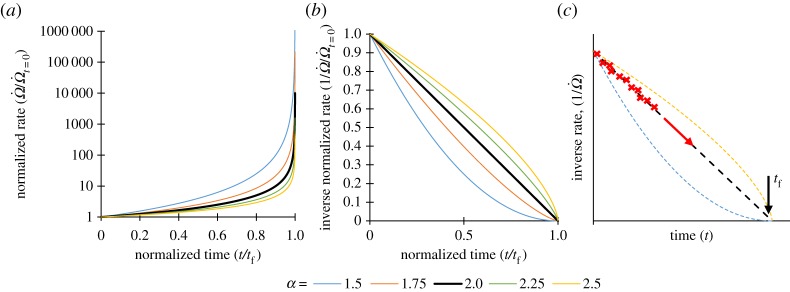
Illustrations of Voight's postulated relationship. (*a*) Damage rate, normalized to the rate corresponding to *t**** ***= 0, against time, normalized to the failure time *t*_f_. Plotted from equation (2.11). (*b*) Inverse normalized damage rate against normalized time, plotted from (*a*) of figure. (*c*) Schematic figure showing inverse rate against elapsed time illustrating the Failure Forecast Method. The red crosses are illustrative of rate data conforming to Voight's postulated behaviour where *α**** ***= 2, the failure time can be estimated by projecting the trajectory of data to the *x*-axis intercept.

Voight's postulated behaviour is illustrated in figure 2 for a range of *α* values. Figure 2*a* shows the familiar increasing rate approaching failure, while figure 2*b* shows the decreasing inverse rate as remnant life expires. It is shown that if *α* = 2 then there is a linear relationship between inverse rate and time, while if *α* *<* 2 the relationship is ‘concave’ and if *α* *>* 2 then it is ‘convex’. Figure 2*c* illustrates graphically the potential utility of combining the empirical behaviour of positive feedback mechanisms with frequent data collection. Plotting the inverse rate against time allows the estimation of the parameters *A* and *α*, which describe the gradient and shape of the inverse rate-time behaviour. The point where inverse rate intersects the *x*-axis is where the rate tends to infinity and so represents the point of criticality; by projecting the best-fitting line the failure time can be estimated. In practice, a solver is used to find best-fitting estimates of *α*, *A* and *t*_f_ of equation (2.11) to the gathered data.

Aside from geophysical processes and the material failure mechanisms of interest in this study, the influence of positive feedback is observable in a range of situations [28–31]. Gravitational waves were recently observed, the identification arising from the near simultaneous detection of a signal with a ‘very specific characteristic’ at two remote locations [32]; this ‘specific characteristic’ is due to positive feedback and it is shown in the electronic supplementary material, §S4 that the signal characteristic *is* Voight's postulated behaviour. Positive feedback mechanisms are common in biological systems, though frequently in nature the increasing rate is limited by a ‘saturating’ influence [28,33]. One example where positive feedback is observed is tumour growth, though lack of consensus on the most accurate growth model [34–36] or availability of sufficiently frequent data means that it is not presently possible to confirm that it conforms to Voight's postulated relationship; there is a clear clinical demand for this type of forecasting [34]. Newly available data from permanently installed sensors may be used to enhance understanding of the system and also to project future points of criticality. It is worth emphasizing that it is possible to project time to criticality not only from the ‘damage’ directly but from indirect symptoms that share the same characteristic behaviour but may be easier to measure; as an example seismic activity of deformation are easily observable metrics that enable the prediction of points of criticality in geophysics [16,19–27]. This is a key benefit of the interpretation of rates as opposed to absolute quantities.

## 3. Experimental demonstration of remnant life prediction for material failure mechanisms

Three damage mechanisms will be used to demonstrate the broad utility of the Failure Forecast Method: fatigue, creep crack growth and volumetric creep. For each mechanism a brief description of the positive feedback will be provided while a more detailed demonstration of how it conforms to Voight's postulated behaviour is given in the electronic supplementary material. Example laboratory data will be presented and used to demonstrate failure time prediction using the Failure Forecast Methodology. All of the results will be discussed collectively in the discussion section, together with further consideration of the appropriate implementation of the methodology and the implications on monitoring strategies.

Two different implementations of the Failure Forecast Method will be demonstrated; the first will assume that *α* *=* 2 and therefore there is inverse proportionality between damage rate and remnant life, the second will not make this assumption and the best-fitting *α* value will be found using the solver. Clearly, in cases where α≠2 the assumption that it is 2 will introduce an error. However, the negative influence of this error may be balanced by the increased stability of failure time estimates resulting from the need to estimate one fewer parameters.

### (a) Fatigue crack growth

A crack in a metal component subject to repeated load cycles will grow according to the well-known, empirical, Paris' Law [37],

3.1 $$\frac{\text{d}a}{\text{d}N}=C\Delta K^{m},$$

where *a* is the crack length and *N* is the number of stress cycles; it is assumed that da/dN may be equated to da/dt and the crack growth rate will be described as a time derivative. *K* is the stress intensity factor,

3.2 $$\Delta K=\Delta \sigma \sqrt{a}Y\left(\frac{a}{W}\right),$$

where Δσ is the range of the stress cycles and *Y* is the dimensionless geometric expression in the stress intensity factor which will be a function of the crack length normalized to component thickness, *W*. Paris’ Law therefore describes the feedback mechanism in fatigue crack growth. As the crack grows the stress concentration increases, leading to an increase in crack growth per stress cycle. Electronic supplementary material, §S2 details further that the form of positive feedback is consistent with Voight's proposed relationship and therefore the Failure Forecast Method should effectively predict remnant life from *in situ* measurements or proxy measurements of crack length.

**Figure 3.**
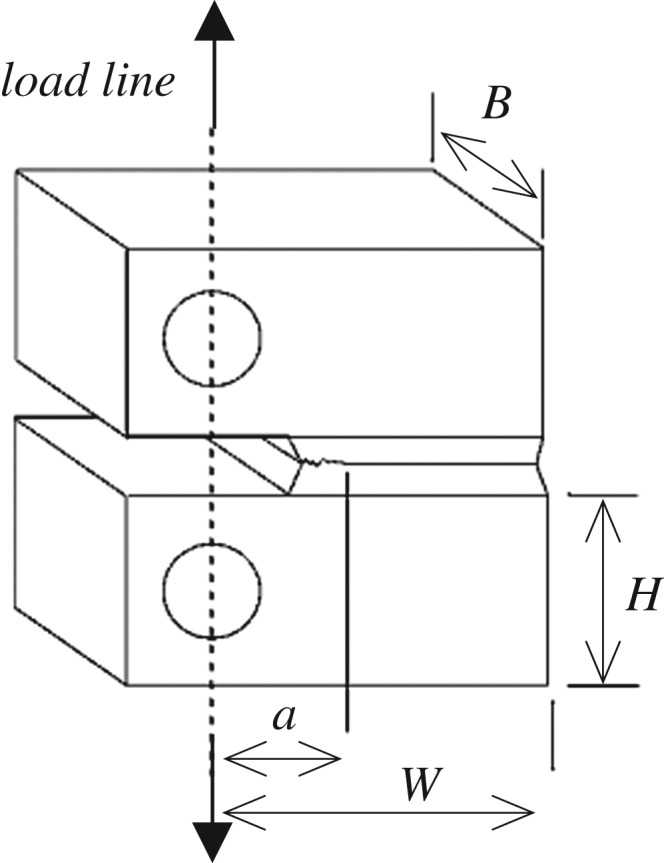
Compact Tension component geometry used for the two fatigue experiments and three creep crack growth experiments in this study.

**Figure 4.**
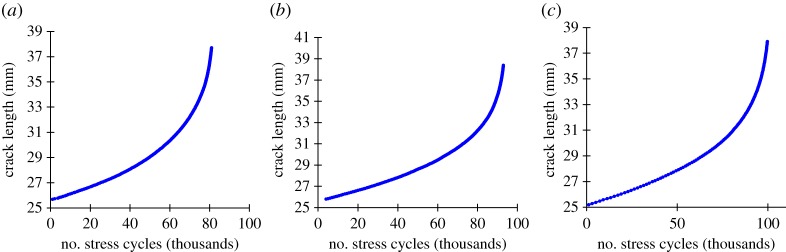
Crack length against number of stress cycles for the (*a*) 80 907, (*b*) 92 923, (*c*) 99 646 cycles to failure experiments tested with conditions listed in table 2. (Online version in colour.)

Data from three fatigue experiments have been taken from the unrelated study [13]; the datasets used were given the identifiers FCG-AR2, FCG-PC2 and FCG-PC1 but are referred to in this study by the failure number of cycles: 80 907, 92 923 and 99 646 cycles respectively. The data is made available in the electronic supplementary material. The standard Compact Tension geometry [38] shown in figure 3 is used for both the fatigue and creep crack growth experiments. Components formed of 316H stainless steel material were tested with the conditions detailed in table 2. The crack length is estimated using the unloading compliance method [13] and is shown in figure 4.

**Table 2.** Test conditions for the three example fatigue experiments.

cycles to rupture	80 907	92 923	99 646
test identifier	FCG-AR2	FCG-PC2	FCG-PC1
		[13]	
pre-conditioning	none	pre-compressed	pre-compressed
temperature		20°C	
applied load		9640 N	
R ratio = minimum load/maximum load		0.1	
initial crack length		nominally 25 mm	
geometry	with reference to figure 3; *B* = 25 mm, *W* = 50 mm, *H* = 30 mm, total side groove depth = 2.25 mm		

Throughout this study, the damage rate (first time derivative) and damage acceleration (second time derivative) are calculated using linear least-squares regression over 10 data points; in this case crack length is the metric of damage.

**Figure 5.**
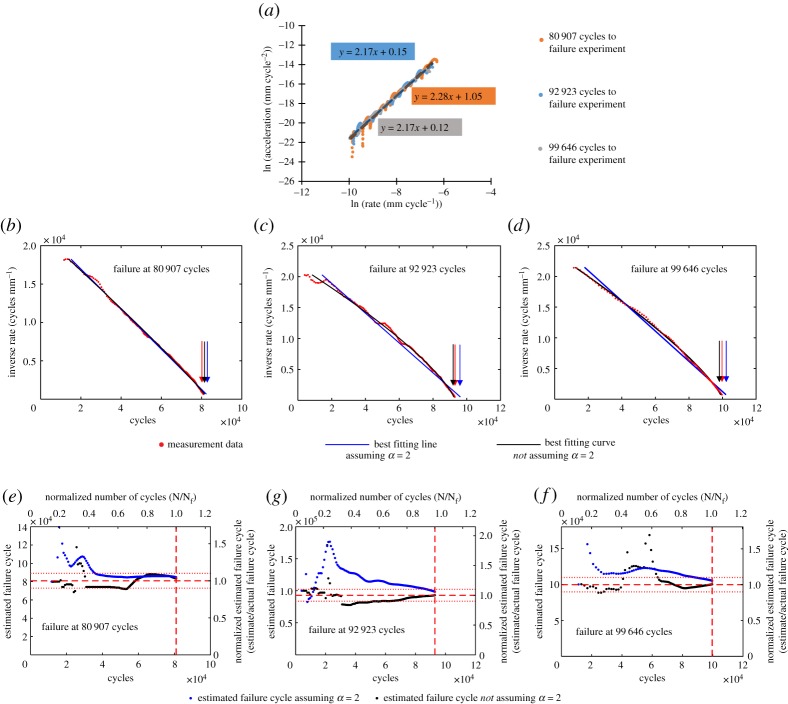
Original data taken from an unrelated study [13]. (*a*) Natural logarithm of crack growth rate (first time derivative) and crack growth acceleration (second time derivative) for the three fatigue crack growth experiments used as examples in this study. Best-fitting least-squares linear relationships shown with dashed lines. The linear relationship shows conformity with Voight's postulated relationship, equation (2.2). The gradient gives the estimated *α* value. (*b–d*) Inverse crack growth rate against number of cycles for the three fatigue experiments. The best-fitting lines are calculated retrospectively for the full dataset. The projected *x*-axis intercept, indicated by the arrows, gives the failure cycle estimate. (*e–g*) Estimated failure cycle calculated throughout the three fatigue experiments using the Failure Forecast Method. The red dashed lines indicate the failure cycle, while the red dotted lines indicate ±10% of the failure cycle. Estimates are only made following the minimum rate.

Plotting the natural logarithm of the first and second time derivatives of crack length offers a convenient means of comparing the experimental data with Voight's postulated behaviour, as shown in figure 5*a*. The linear relationship indicates agreement with the postulation and further the gradient gives the value of *α*; the gradient is found to be between 2.17 and 2.28 for the three experiments, which is consistent with the expectation that it is frequently approximately 2.

The Failure Forecast Method is carried out for the experimental data. To illustrate this process, the inverse crack growth rate is plotted against experiment time in figure 5*b–d*. As all *α* values are greater than 2 then the best-fitting curves are seen to be slightly convex. The lines described by the best-fitting parameters of equation (2.11), calculated retrospectively for the full dataset, are shown. Both solutions where *α* is assumed to be 2 and where *α* is found as an additional best-fitting parameter are included. In practice, with each new data point the process is repeated for all previous data and the best-fitting parameters, including the projected failure time, are refined.

The estimated failure time solutions from this methodology are shown in figure 5*e–g*. The failure time estimates assuming both *α* is 2 and where *α* is found by the solver are plotted to compare the solutions.

### (b) Creep crack growth

The crack growth rate of a cracked component subject to static load at high temperature may be described by the C* relation [9]

3.3 $$\frac{\text{d}a}{\text{d}t}=D{C^{*}}^{\phi },$$

where *D* is a constant, *ϕ* is a constant equal to (n−1)/n, where *n* is the Norton stress exponent and C* is the ‘steady state creep crack tip parameter’ which is shown in the electronic supplementary material, §S3 to be a function of crack length *a* using reference stress methods [9,39,40]. The C* relationship therefore describes the positive feedback mechanism. The increasing crack length increases the stress concentration and also the effective stress on the remaining cross section.

Three datasets taken from the ODIN MatDB database are used in this study [14,41,42]. The experiments used the same Compact Tension geometry illustrated in figure 3 and test conditions shown in table 3; the different components will be referred to by the test time to failure; 65 h [14], 504 h [41] and 1068 h [42].

**Table 3.** Test conditions for the three example creep crack growth experiments.

time to rupture	65 h	504 h	1068 h
MATDB test identifier	CT041H [14]	CT10 [41]	CT045H [42]
temperature	600°C	600°C	600°C
initial stress	4100 N	3030 N	2700 N
environment	hydrogen	air	hydrogen
initial crack length	13.3 mm	12.9 mm	12.8 mm
geometry	with reference to figure 3: *B* = 12.5 mm, *W* = 25 mm, *H* = 15 mm, total side groove depth = unspecified but assumed to be 2.5 mm		

**Figure 6.**
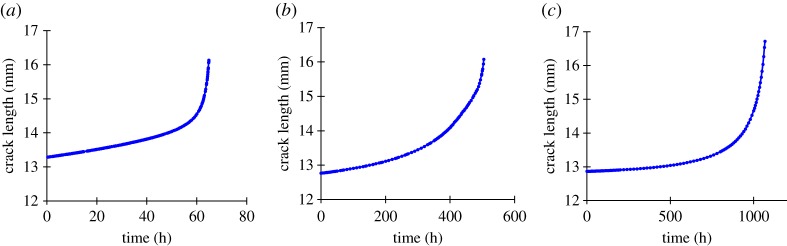
Crack length against time for the (*a*) 65, (*b*) 504, (*c*) 1068 h to failure experiments tested with conditions listed in table 3. (Online version in colour.)

The crack length was calculated from load line displacement data and is shown in figure 6.

**Figure 7.**
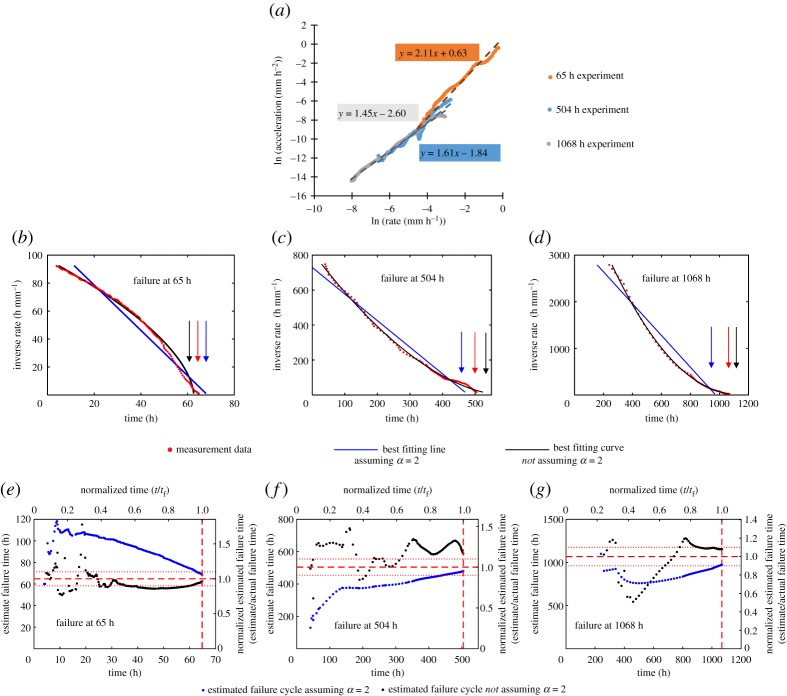
Original data taken from ODIN MatDB database [14,41,42]. (*a*) Natural logarithm of crack growth rate (first time derivative) and crack growth acceleration (second time derivative) for the three creep crack growth experiments used as examples in this study. Best-fitting least-squares linear relationships shown with dashed lines. The linear relationship shows conformity with Voight's postulated relationship, equation (2.2). The gradient gives the estimated *α* value. (*b–d*) Inverse crack growth rate against time for the three fatigue experiments. The best-fitting lines are calculated retrospectively for the full dataset. The projected *x*-axis intercept, indicated by the arrows, gives the failure time estimate. (*e–g*) Estimated failure time calculated throughout the three fatigue experiments using the Failure Forecast Method. The red dashed lines indicate the failure time, while the red dotted lines indicate ±10% of the failure time. Estimates are only made following the minimum rate.

The natural logarithm of the first and second time derivatives of crack length are shown in figure 7*a*. The approximately linear fit indicates agreement with Voight's postulated relationship. On this occasion there is a range of gradients, and therefore best-fitting *α* values, from 1.44 to 2.11.

The inverse crack growth rate against time data is shown in figure 7*b–d* for each of the three experiments. The best-fitting linear relationship (i.e. assuming *α* is 2) and nonlinear relationship (not assuming *α* is 2) are plotted retrospectively for the full dataset. It can be observed that due to the 65 h experiment having a best-fitting *α* value greater than 2 it has a convex form, while the 504 and 1068 h experiments have an *α* value less than 2 and so the relationships are concave. Figure 7*e–g* show the estimated failure time obtained from the Failure Forecast Method; the procedure is repeated at each new data point for all previous data and the failure time estimates refined.

### (c) Volumetric creep

Voight uses creep of metal alloys in his original proposition as an example of a well-known failure mechanism that can be shown to be consistent with his postulated relationship. His argument is updated here with reference to more recent literature. Creep strain rate is symptomatic of creep damage, which constitutes a range of different mechanisms, and it is understood that many of the most dominant mechanisms are strain controlled. It has previously been argued that the approximate relationship describing creep strain rate behaviour is [16,43–45],

3.4 $$\frac{\text{d}\varepsilon }{\text{d}t}={\left(\frac{\text{d}\varepsilon }{\text{d}t}\right)}_{0}\text{ex}{\text{p}}^{A\varepsilon },$$

where *ε* is true strain, the subscript 0 denotes a reference strain rate, which occurs at or after the minimum creep rate following ‘primary’ [46] creep and *A* is a constant for the operating conditions and material properties. It was also shown [16] by differentiating this function that it was consistent with Voight's postulated relationship, equation (2.2). The reason for conformity with Voight is that creep is a positive feedback damage mechanism: an increase in strain leads to an increase in damage, which leads to an increase in strain rate. For, completeness, differentiating equation (3.4) with respect to *ε* shows that

3.5 $$\frac{\text{d}(\text{d}\varepsilon /\text{d}t)}{\text{d}\varepsilon }=A{\left(\frac{\text{d}\varepsilon }{\text{d}t}\right)}_{0}\text{ex}{\text{p}}^{A\varepsilon }=A\left(\frac{\text{d}\varepsilon }{\text{d}t}\right),$$

and therefore satisfies the condition of equation (2.6) with *α* = 2. This study does not seek to justify the exact form of equation (3.4) but rather relies on the assumption of underlying positive feedback.

**Figure 8.**
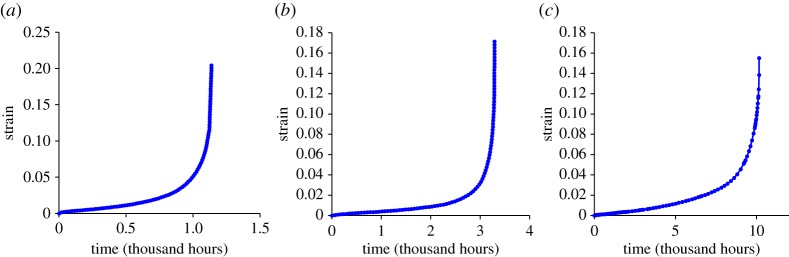
Creep strain against time for the (*a*) 1138, (*b*) 3289, (*c*) 10 115 h to failure experiments tested with conditions listed in table 4. (Online version in colour.)

To demonstrate the Failure Forecast Method for creep damage, three datasets have again been taken from the ODIN MatDB database [15,47,48]. Each constant load experiment used a cylindrical uniaxial specimen of 14MoV6-3 steel, with a range of test conditions given in table 4. The three experiments will be referred to by the time to rupture; 1138, 3289 and 10 115 h. The raw strain against time data is shown in figure 8.

**Table 4.** Test conditions for the three example creep experiments.

time to rupture	1138 h	3289 h	10 115 h
MatDB test identifier	BLE004 [47]	BLE012 [48]	BLE013 [15]
temperature	600°C	600°C	550°C
initial stress	140 MPa	110 MPa	165 MPa

**Figure 9.**
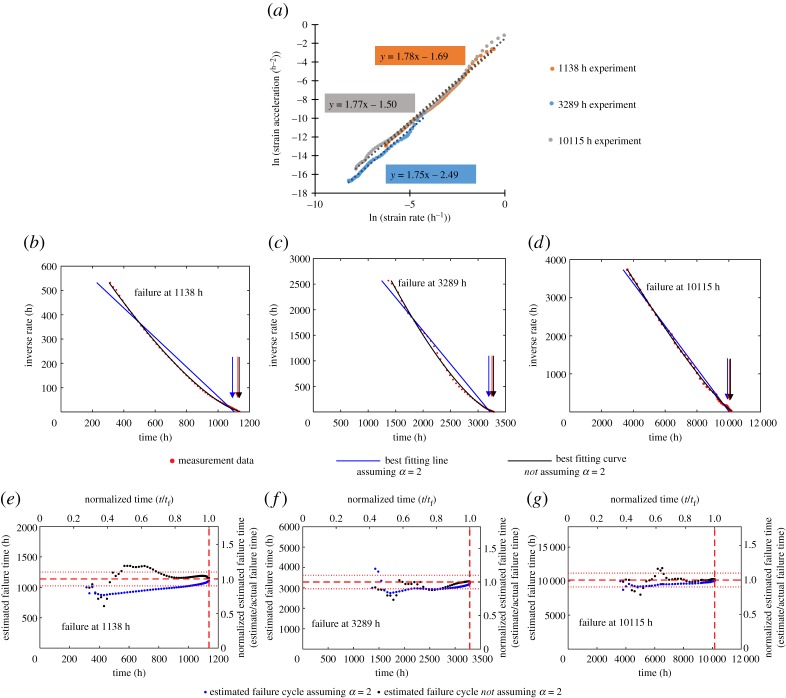
Original data taken from ODIN MatDB database [15,47,48]. (*a*) Natural logarithm of strain rate (first time derivative) and strain acceleration (second time derivative) for the three creep experiments used as examples in this study. Best-fitting least-squares linear relationship shown with dashed lines. The linear relationships shows conformity with Voight's postulated relationship, equation (2.2). The gradient gives the estimated *α* value. (*b–d*) Inverse strain rate against time for the three fatigue experiments. The best-fitting lines are calculated retrospectively for the full dataset. The projected *x*-axis intercept, indicated by the arrows, gives the failure time estimate. (*e–g*) Estimated failure time calculated throughout the three creep experiments using the Failure Forecast Method. The red dashed lines indicate the failure time, while the red dotted lines indicate ±10% of the failure time. Estimates are only made following the minimum rate.

The first and second time derivatives are calculated from the experimental data and the ‘primary’ creep data prior to the minimum creep rate is discarded. Figure 9*a* is plotted to demonstrate the agreement with Voight's postulated behaviour. The gradient of each line, and therefore the best-fitting *α* value, is between 1.75 and 1.80.

Figure 9*b–d* shows the inverse strain rate against time behaviour for each of the three experiments. As the *α* values were found to be similar across the three experiments, the curvature is similarly concave. The Failure Forecast Method is then carried out on the creep data, as shown in figure 9*e–g*. Due to the prolonged ‘primary’ stage, failure estimates cannot commence until a significant portion of life is already exhausted.

## 4. Discussion

Similar features are observable for each of the different mechanisms and so it is appropriate to discuss them collectively.

In general, where *α* is not assumed to be 2 the failure time estimates are within 10% of the true failure time from early on in the experiment. This is significant as the estimates are based purely on the observed response of the component, without any peripheral information on material properties or operating conditions. Estimates are made only with the data collected up until that point and are seen to be accurate at a stage when changes in rates are barely observable from the damage versus time plots. It is evident that in cases where *α* is less than 2 the failure times are in general overestimated, a consequence of the concave inverse rate versus time relationship. In practice the component will fail before the rate reaches infinity, or equivalently inverse rate reaches zero, and so the failure time estimate will be overestimated; the error is exacerbated in the cases where *α* is less than 2 as the inverse rate-time relationship is increasingly shallow approaching failure. This issue is particularly evident in figure 7*c*,*d*.

The estimates where *α* is assumed to be 2 are in general more stable, as most evident in figure 5*a*,*c*. As the value for *α* is already assumed, one fewer parameter needs to be estimated which could otherwise potentially introduce further variability. This benefit may be more apparent in data from less controlled situations as solutions are less likely to be influenced by fluctuations in data. Due to the inaccuracy of the assumption, however, systematic errors are clearly evident in these solutions; when *α* *<* 2 the failure times are underestimated while when *α* > 2 the failure times are overestimated. This is due to the change in the gradient of the inverse rate over time. In the concave case, the best-fitting straight line is initially steep and so the estimated failure time where the projected line meets the *x*-axis occurs prior to the true failure time. In the convex case, the best-fitting straight line is initially shallow and is therefore projected past the true failure time. As more data is gathered the gradient is refined and so the systematic error is reduced.

The choice between whether to assume *α* is or is not 2 is clearly determined by the particular situation. Firstly, *α* should be estimated to ascertain how accurate the assumption is. Additionally, the choice of approach is determined by whether a more stable solution is required due to fluctuations in the measurement data. There may also be merit in evaluating and comparing both solutions, with the observation that frequently the two failure time estimates bracket the true failure time. It is to be emphasized that there are very many ways of projecting the anticipated failure time based on Voight's postulated relationship—in the present study a basic framework was presented for clarity of demonstration. The process may be improved, for example, with more sophisticated processing of data, statistical models, data segmentation and the use of additional data or information.

The successful implementation of the methodology relies on accurate and reliable rate measurements which are difficult to obtain from infrequent inspection type measurements. The confidence interval associated with a rate estimate is proportional to the square root of the time interval between successive measurements, as demonstrated in the electronic supplementary material, §S1; random measurement uncertainty can therefore be overcome by increasing the frequency of readings. For practical situations, permanently installed sensors are necessary so that the frequency of readings is sufficient to achieve the required certainty in rate measurements. Additionally, an increased number of measurements leads to improved solutions when parameters are best-fitted to available data.

Any estimate of failure time is based on the assumed continuation of the present operating conditions; a future change in conditions will undermine the estimate, an increase in load will cause apparently premature failure while a decrease in load will cause the estimate to be conservative. Fortunately, the Failure Forecast Method can be started at any point which means that if there has been a change in conditions then previous data can be discarded and an updated solution reflecting the new conditions can be calculated. Updating the solution also offers a means for accounting for history-dependent processes, such as dislocation density accumulation, slip accumulation, or back-stress development, or to account for subtle evolution of the damage mechanism.

The failure criterion used in this study is taken to be the point of eventual rupture at the present operating conditions. Frequently in structural integrity standards (*API 579-1/ASME FFS-1* (API/ASME, 2007)) the safe operating life is dictated by the ability of the component to withstand an exceptional event as opposed to ultimately failing under usual operating conditions; the methodology presented in this study may require some refinement to assess the robustness against a given exceptional event. Nonetheless, it is believed that the rupture life is a valuable supplementary piece of information; as an example, a great deal of investment is made in replica metallography for the evaluation of volumetric creep damage, despite the risk of life-limiting damage being more usually associated with welds or stress-concentrations [9,49].

**Figure 10.**
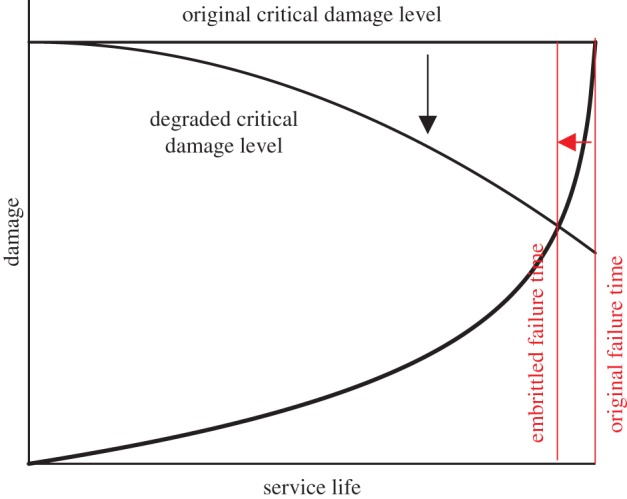
Schematic illustration showing that possible embrittlement through the service life of a component can cause a reduction in the critical damage level. Fortunately, the reduction in the life of the component is expected to be modest. Illustration adapted from [7].

The assumed failure criterion that the damage rate will reach infinity at failure should be considered for each application. Many structural engineering components are designed to fail in a ductile ‘leak-before-break’ rather than brittle manner. In ductile failure the damage will propagate through the whole of the component before a critical damage level which causes spontaneous failure is reached; in such situations the rate of change of damage is expected to be very large at failure. Brittle failure is usually designed against due to its inherently unpredictable nature. However, it is noted that with extended service exposure, embrittlement phenomena can occur, reducing the toughness of the component so that the critical damage level is sufficiently low to cause brittle failure. This is represented schematically in figure 10 and discussed in [7]. Fortunately, however, as the rate of increase in damage accelerates towards failure, even a significant decrease in the critical damage level is expected to result in a relatively modest reduction in life, as shown in figure 10. Nonetheless, the likely risk of brittle failure should be considered for each application.

The positioning and area coverage of permanently installed sensors is critical; each application must be considered on an individual basis to ensure that sensors are placed so that the progress of damage is effectively captured. In the example of crack growth, the analysis is applicable only to the terminal stages of component life where a crack is already present; the location of likely crack initiation points or existing cracks must be known in order to install sensors in the appropriate locations. Likewise, in the case of creep, the sensor must be positioned in a location which captures the life-limiting maximum damage level, so that failure time estimates are conservative. Too great an area coverage can also be problematic; if the damage is particularly localized and the sensor averages damage over a large area, then the maximum extent of the damage may be underestimated. An example where this is significant is in Type IV creep failure of weldments. Damage is localized to a narrow region in the heat-affected zone and so failure appears brittle occurring with little deformation when in fact large strain is evident locally in the heat-affected zone; a sensor with a small gauge length is therefore advantageous [50]. As the Failure Forecast Method can be started at any point then a sensor may be installed once areas of interest have become apparent.

A key benefit in the interpretation of relative *rates of change* as opposed to ‘absolute’ measures of damage severity is the flexibility it affords in the measured damage metric; particularly in biomedical applications there may be metrics that are symptomatic of the underlying positive feedback mechanism that will conform to the same rate behaviour and are therefore interpretable in the same way. The damage metric can therefore be relatively arbitrary provided that it is symptomatic of the damage feedback mechanisms; inversion of sensor outputs to provide ‘absolute’ damage measurements may not be necessary. If *z* is a measurement output that is related to the sought damage parameter, *Ω*, then *z* = *z*(*Ω*) and the rate of change of measurement output would be,

4.1 $$\frac{\text{d}z}{\text{d}t}=\frac{\text{d}z}{\text{d}\Omega }\frac{\text{d}\Omega }{\text{d}t},$$

where it will be assumed that dΩ/dt=f(Ω) is a positive feedback mechanism as before. Whether the measurement output is subsequently consistent with Voight's postulated relationship, equation (2.2), is determined by the sensitivity dz/dΩ and a criterion equivalent to equation (2.6) would need to be demonstrated,

4.2 $$\frac{\text{d}(\text{d}z/\text{d}t)}{\text{d}z}=A{\left(\frac{\text{d}z}{\text{d}t}\right)}^{\alpha -1}.$$

This demonstration may not be feasible, especially if the sensitivity, *z* = *z*(*Ω*), is not known explicitly. However, some understanding of what is expected from a measurement output is gained by considering the example where the measurement output is given by the general expression *z* = *Ω^β^*. If *β* *=* 1 then the measurement is proportional to damage. If *β* < 1 then the sensitivity of the measurement decreases with damage, as would occur, for example if saturation begins in the range of interest; if *β* > 1 then the sensitivity increases with damage. The sensitivity is given by

4.3 $$\frac{\text{d}z}{\text{d}\Omega }=\beta \Omega^{\beta -1},$$

and therefore the time response is described by,

4.4 $$\frac{\text{d}z}{\text{d}t}=\beta \Omega^{\beta -1}\frac{\text{d}\Omega }{\text{d}t}.$$

As the damage level and rate of damage increase monotonically with time then provided *β* > 1 the rate of measurement output must also increase monotonically with time and therefore proximity to failure, the acceleration is amplified by the increasing measurement sensitivity. On the other hand if *β* < 1, then the decreasing sensitivity will compete with the increasing rate of damage and the measurement output is unlikely to be consistent with Voight's postulated relationship.

In some situations a metric of rate is more easily obtained than a metric of absolute damage; in such situations interpreting rates directly offers a significant advantage. The acoustic emission count rate per load cycle, $\text{d}N_{AE}/\text{d}N$, is proportional to the rate of change of crack length, da/dN [51]. The Failure Forecast Method enables direct interpretation of the count rate without the need to invert crack length; this is a similar suggestion to the established methodology of exploiting easily measurable seismological data for predicting geophysical events.

Voight's proposed relationship and remnant life prediction method is established in geophysics and is now proposed for structural integrity assessment; it is believed that the behaviour is likely to be found among numerous diverse examples of systems controlled by positive feedback beyond this. The ever advancing development and popularity of remote sensors and wireless connectivity may result in further applications of the Failure Forecast Method. Causal loop diagrams may help identify and model complex situations where positive feedback is present and identify possible measureable symptoms; they may also help identify constraints to the positive feedback and where negative feedback is present which may alter the observed response away from Voight's postulation.

## 5. Conclusion

A number of damage mechanisms share the common characteristic of an increasing rate of damage with proximity to failure. This is a consequence of positive feedback: the presence of damage compromises the components capacity to sustain load and therefore the rate of damage increases; the rate of change will accelerate until eventual failure. Voight proposed an empirical relationship that describes this behaviour which can be used to estimate remnant life in what is known as the Failure Forecast Method. The methodology uses *in situ* data to predict failure in real-time, based on observed component response, without relying on estimates of operating condition or material properties.

In this study fatigue, creep crack growth, and volumetric creep have been used as illustrative examples; they have been shown to be positive feedback mechanisms, and are consequently consistent with Voight's postulated relationship and compatible with the Failure Forecast Method. It has been demonstrated using published experimental data available in the open literature that projected failure times are accurate from shortly after the start of data collection.

Voight's proposed relationship and remnant life prediction method is established in geophysics and is now proposed for structural integrity assessment; it is believed that the behaviour is likely to be found among numerous diverse examples of positive feedback beyond this. The ever advancing development and popularity of remote sensors and wireless connectivity may result in further applications of the Failure Forecast Method.

The successful implementation of the methodology relies on accurate and reliable rate measurements which are difficult to obtain from infrequent inspection type measurements. The confidence interval associated with a rate estimate is proportional to the square root of the time interval between successive measurements; random measurement uncertainty can therefore be overcome by increasing the frequency of readings. For practical engineering situations permanently installed sensors are necessary so that the frequency of readings is sufficient to achieve the required certainty in rate measurements. Additionally, an increased number of measurements leads to improved solutions when best fitting parameters to data points.

The interpretation of changes in rates, as opposed to absolute damage, affords flexibility in the choice of monitoring technique. The metric which is to be used should be symptomatic of the positive feedback mechanism but does not necessarily need to be a direct measurement of damage severity. Monitoring techniques such as Acoustic Emission provide measurements which are indicative of rate rather than absolute damage and therefore may be used for the Failure Forecast Method without any need for inversion.

The interpretation of rates of change offers a paradigm shift in assessing the risk of critical events. Increasingly ubiquitous permanently installed sensors can be used to gather near-continuous data on symptoms of damage which may be interpreted for remnant life estimation, in real-time, in a way which is specific to the unique system under investigation.

## Supplementary Material

Supplementary Text

## Supplementary Material

Supplementary Data - Fatigue

## Data accessibility

The data used in this study is publically available and cited or is available in the electronic supplementary material.
