# Supplementary material for: Rate-based structural health monitoring using permanently installed sensors
Source: Proc Math Phys Eng Sci. 2017 Sep 13;473(2205):20170270. doi: 10.1098/rspa.2017.0270 (PMC5627375; doi:10.1098/rspa.2017.0270)
Supplement: Supplementary Text [file rspa20170270supp1.pdf]

Supplementary Materials for

Rate based structural health monitoring using permanently installed sensors

Proceedings of the Royal Society A: Mathematical, Physical & Engineering Sciences

Joseph Corcoran

correspondence to: joseph.corcoran07@imperial.ac.uk

Supplementary Text

S1. Uncertainty of linear rate estimates calculated from a series of measurements
S2. Demonstration that fatigue is a positive feedback mechanism that is consistent with Voight's postulated relationship
S3. Demonstration that creep crack growth is a positive feedback mechanism that is consistent with Voight's postulated relationship
S4. Demonstration that the gravitational waves from inspiraling black holes behave according to Voight's postulated relationship

Supplementary Text

S1. Uncertainty of linear rate estimates calculated from a series of measurements

This section presents the analysis required to calculate the expected uncertainty of a rate estimate calculated from a series of points, each with an associated random uncertainty. This is crucial in order to evaluate the expected performance of rate measurement systems and also to inform measurement strategy.

The objective of this section is to derive an expression for the uncertainty of the gradient estimate for a given confidence level; for example $b \pm \Delta b$ at 95% confidence interval. To do so we start with the model that N measurements are taken at a measurement repetition frequency of f_m over a total length of time, t_m, each measurement has a standard error of σ as shown in Figure S1.

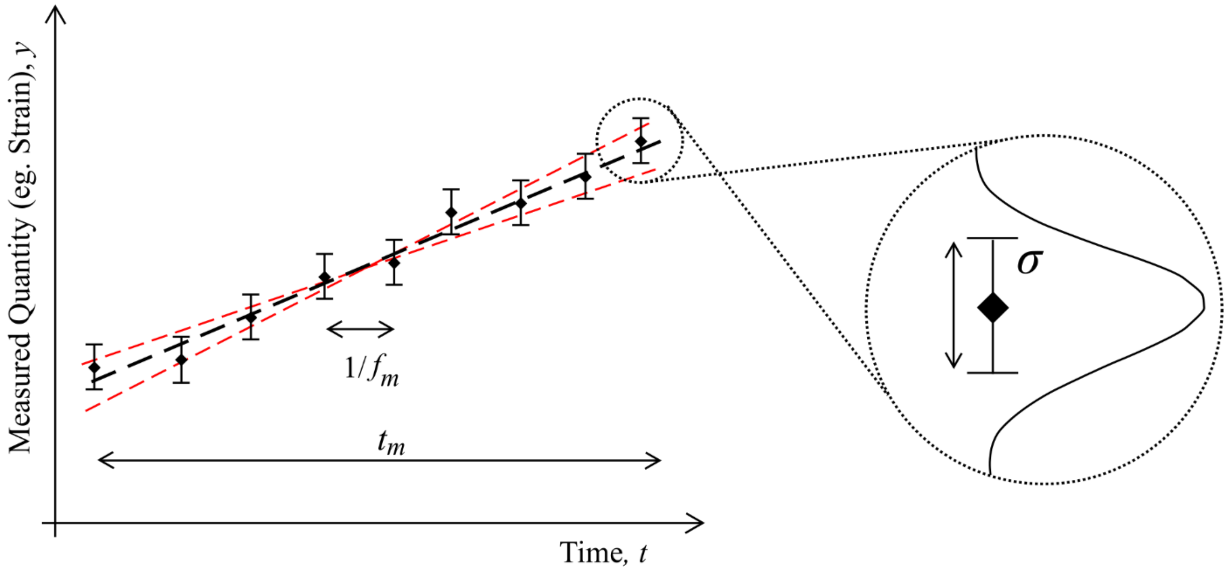

Figure S1: Schematic showing an estimate of a gradient from a series of measurements, each with a given uncertainty. This section is dedicated to evaluating the uncertainty of the gradient estimate, illustrated by the red dashed lines.

The standard error, S, of the straight line fit is the square root of the sum of the squares of the deviations from the straight line,

$$S^2 = \sum_{i=1}^{N} (y_i - a - bt_i)^2 \tag{S1}$$

where (t_i, y_i) are measurement pairs. The standard error of the gradient is related to the individual measurement standard deviation, σ, according to,

$$S^2 = (N-2)\sigma^2 \tag{S2}$$

It can be shown (*55*) that the variance of the gradient estimate is given by,

$$\sigma_{grad}^2 = \frac{S^2}{(N-2)\sum_{i=1}^{N}(t_i - \bar{t})^2} = \frac{\sigma^2}{\sum_{i=1}^{N}(t_i - \bar{t})^2} \tag{S3}$$

where $\bar{t}$ is the mean time of the measurement $\approx t_m/2$. If the individual thickness readings are normally distributed, the slope follows a 't' distribution with $(N-2)$ degrees of freedom. However, we are interested in large numbers of readings so the 't' distribution is approximately normal (for N>25, the error is under 10%). Therefore,

$$\Delta b = \sigma_{grad} P \tag{S4}$$

P is the ‘t score’ which is a function of N and the desired level of confidence. However, if N is large then P becomes independent of N (the distribution becomes normal as discussed above) and P is as given in Table S1.

Table S1: Values of P for at different confidence levels.

Confidence Level (%)	*P*
90	*1.645*
95	*1.960*
99	*2.576*

Now,

$$t_i = (i-1)\Delta t \tag{S5}$$

and,

$$\bar{t} = \frac{(N-1)\,\Delta t}{2} \tag{S6}$$

so,

$$\sum_{i=1}^{N} (t_i - \bar{t})^2 = \Delta t^2 \sum_{i=1}^{N} \left((i-1) - \frac{(N+1)^2}{2} \right)^2 \ . \tag{S7}$$

Expanding gives,

$$\sum_{i=1}^{N} (t_i - \bar{t})^2 = \Delta t^2 \sum_{i=1}^{N} \left(i^2 - (N+1)i + \frac{(N+1)^2}{4} \right)^2 \ . \tag{S8}$$

Using standard expressions for sums of series in i^2 and i gives,

$$\sum_{i=1}^{N} (t_i - \bar{t})^2 = \Delta t^2 \left(\frac{2N^3 + 3N^2 + N}{6} - \frac{N(N+1)^2}{2} + \frac{N(N+1)^2}{4} \right) \tag{S9}$$

which reduces to,

$$\sum_{i=1}^{N} (t_i - \bar{t})^2 = \frac{\Delta t^2}{12} (N^3 - N) \tag{S10}$$

Equation S3 into Equation S4 gives,

$$\Delta b = \sigma_{grad} P = \frac{\sigma P}{\sqrt{\sum_{i=1}^{N} (t_i - \bar{t})^2}} \tag{S11}$$

And then Equation S10 into S11 gives,

$$\Delta b = \frac{\sigma P \sqrt{12}}{\Delta t \sqrt{N^3 - N}} \approx \frac{\sigma P \sqrt{12}}{\Delta t N^{3/2}} \tag{S12}$$

as N is large. Using the terms introduced in Figure S1, $\Delta t = 1/f_m$ and $N = T f_m$ so,

$$\Delta b = \frac{\Delta t^{1/2}\sigma P\sqrt{12}}{T^{3/2}} \tag{S13}$$

For a given elapsed time period available to take measurements the uncertainty is proportional to the square root of the time interval between measurements.

S2. Demonstration that fatigue is a positive feedback mechanism that is consistent with Voight's postulated relationship

Following from Equation (13) in the main text. There are different Y functions for given typical geometries which can be found in numerous reference tables (*39*). An example is for the 'Compact Tension' standard geometry (*39*), with dimensions shown in Figure 3 experiencing constant load amplitude cycles of ΔP,

$$\Delta K = \frac{\Delta P}{B\sqrt{W}} \frac{\left(2 + \left(\frac{a}{W}\right)\right)}{\left(1 - \left(\frac{a}{W}\right)\right)^{\frac{3}{2}}} \left(0.886 + 4.64\left(\frac{a}{W}\right) - 13.32\left(\frac{a}{W}\right)^2 + 14.72\left(\frac{a}{W}\right)^3 - 5.6\left(\frac{a}{W}\right)^4\right). \tag{S14}$$

which is valid for $a/W > 0.3$. By combining Equation (12) and (S14),

$$\frac{d\left(\frac{a}{W}\right)}{dN} = \frac{C}{W}\left\{\frac{\Delta P}{B\sqrt{W}} \frac{\left(2 + \left(\frac{a}{W}\right)\right)}{\left(1 - \left(\frac{a}{W}\right)\right)^{\frac{3}{2}}} \left(0.886 + 4.64\left(\frac{a}{W}\right) - 13.32\left(\frac{a}{W}\right)^2 + 14.72\left(\frac{a}{W}\right)^3 - 5.6\left(\frac{a}{W}\right)^4\right)\right\}^m \tag{S15}$$

If the group of constants $\frac{C}{W}\left(\frac{\Delta P}{B\sqrt{W}}\right)^m$ is κ then Equation (S15) can be written,

$$\frac{d\left(\frac{a}{W}\right)}{dN} = \kappa f\left(\frac{a}{W}\right) \tag{S16}$$

It is therefore a positive feedback damage mechanism. To negate the dependence on the group of constants, κ, the relationship can be normalised to an initial crack length a_0.

$$\frac{\frac{d\left(\frac{a}{W}\right)}{dN}}{\frac{d\left(\frac{a_0}{W}\right)}{dN}} = \frac{f\left(\frac{a}{W}\right)}{f\left(\frac{a_0}{W}\right)} \tag{S17}$$

It is then possible to plot normalised rate against normalised crack length, as shown in Figure S2. If m is assumed to be 3 (*54*) and $a_0/W = 0.3$.

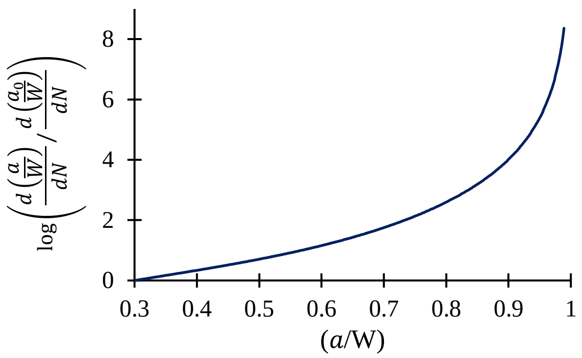

Figure S2: Normalised crack growth rate as a function of normalised crack length. Plotted from Equation (S17).

For compatibility with the Voight's postulation $\frac{d^2\Omega}{dt^2} = A\left(\frac{d\Omega}{dt}\right)^{\alpha}$ and the Failure Forecast Method fatigue crack growth must fulfil the requirement,

$$\frac{df(\Omega)}{d\Omega} = Af(\Omega)^{\alpha-1} = A\left(\frac{d\Omega}{dt}\right)^{\alpha-1} \tag{S18}$$

If the damage state is determined by normalised crack length a/W then the relationship shown in Figure S2 can be numerically differentiated to show good agreement with the form of equation equivalent to Equation (6) as $f\left(\frac{a_0}{W}\right)$ is a constant and can be incorporated into the A parameter.

$$\frac{d\left[\frac{f\left(\frac{a}{W}\right)}{f\left(\frac{a_0}{W}\right)}\right]}{d\left(\frac{a}{W}\right)} = A\left[\frac{f\left(\frac{a}{W}\right)}{f\left(\frac{a_0}{W}\right)}\right]^{\alpha-1} \tag{S19}$$

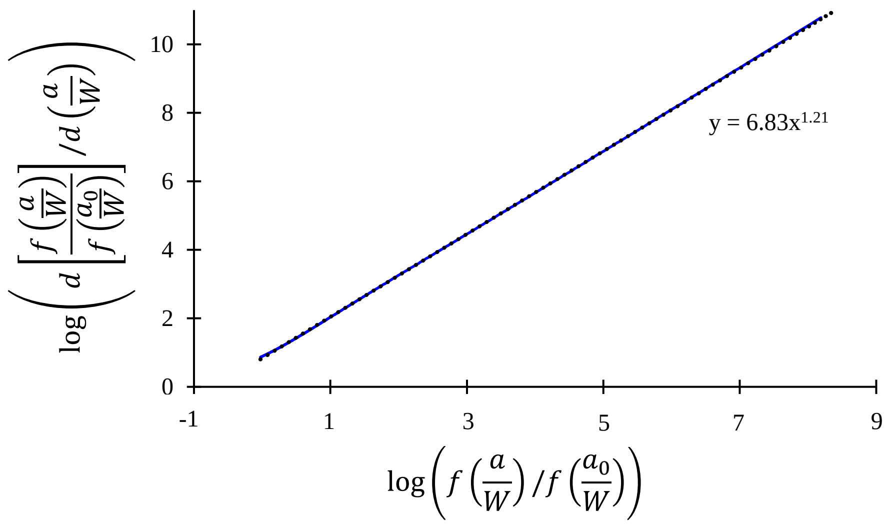

Figure S3: Normalised crack growth rate differentiated with respect to normalised crack length against normalised crack length. The power law relationship fulfils the criterion of Equation 4 indicating that it is consistent with Voight's postulated relationship, Equation 2.

The best fitting exponent is 1.21 and therefore α is expected to be approximately 2.21.

S3. Demonstration that creep crack growth is a positive feedback mechanism that is consistent with Voight's postulated relationship

Following from Equation (14) in the main text, C^* is the steady state creep crack tip parameter which is equal to,

$$C^* = \sigma_{ref}\ \dot{\varepsilon_{ref}} \left(\frac{K}{\sigma_{ref}}\right)^2 \qquad \text{(S20)}$$

K is the stress intensity factor equivalent to that for fatigue which, by replacing load range and stress intensity factor range in Equation (S14) by load and stress intensity factor respectively, gives,

$$K = \frac{P}{B\sqrt{W}} \frac{\left(2+\left(\frac{a}{W}\right)\right)}{\left(1-\left(\frac{a}{W}\right)\right)^{\frac{3}{2}}} \left(0.886 + 4.64\left(\frac{a}{W}\right) - 13.32\left(\frac{a}{W}\right)^2 + 14.72\left(\frac{a}{W}\right)^3 - 5.6\left(\frac{a}{W}\right)^4\right) \qquad \text{(S21)}$$

$$= \frac{P}{B\sqrt{W}} f\left(\frac{a}{W}\right).$$

σ_{ref} is the reference stress and $\dot{\varepsilon_{ref}}$ is the creep strain rate at the current reference stress and strain rate which may be calculated using,

$$\dot{\varepsilon_{ref}} = A\sigma_{ref}^n \qquad \text{(S22)}$$

Therefore,

$$C^* = A\sigma_{ref}^{n+1}\left(\frac{K}{\sigma_{ref}}\right)^2 \tag{S23}$$

and subsequently,

$$C^* = A\sigma_{ref}^{n-1}K^2 \tag{S24}$$

σ_{ref} is defined as

$$\sigma_{ref} = P\frac{\sigma_y}{P_{LC}} \tag{S25}$$

Where σ_y is the yield stress, P is the applied load and P_{LC} is the plastic collapse load for the remaining cross section which can be calculated using a function specific to the geometry and the normalised crack length. For a Compact Tension, 'C(T)', specimen,

$$\frac{\sigma_y}{P_{LC}} = \frac{1}{BW\left[g\left(\frac{a}{W}\right)\right]} = \frac{1}{BW\left[-\left(1-\frac{2}{\sqrt{3}}\left(\frac{a}{W}\right)\right)+\sqrt{(1+\frac{2}{\sqrt{3}})(1+\frac{2}{\sqrt{3}}\left(\frac{a}{W}\right)^2)}\right]} \tag{S26}$$

Inserting (S26) into (S25) and rewriting for brevity,

$$\sigma_{ref} = P\frac{\sigma_y}{P_{LC}} = \frac{P}{BW\left[g\left(\frac{a}{W}\right)\right]} \tag{S27}$$

Equations (S21) and (S27) can then be inserted into (S20) and subsequently into (14) to give,

$$\frac{d(\frac{a}{W})}{dt} = \frac{D}{W}\left\{A\left(\frac{P}{BW\left[g\left(\frac{a}{W}\right)\right]}\right)^{n-1}\left(\frac{P}{BW}\sqrt{W}\sqrt{\left(\frac{a}{W}\right)}f\left(\frac{a}{W}\right)\right)^2\right\}^{\phi} \tag{S28}$$

Gathering constants,

$$\frac{d(\frac{a}{W})}{dt} = \frac{D}{W}\left\{A\left(\frac{P}{BW}\right)^{n-1}\left(\frac{P}{BW}\sqrt{W}\right)^2\right\}^{\phi}\left\{\left(\frac{1}{\left[g\left(\frac{a}{W}\right)\right]}\right)^{n-1}\left(\sqrt{\left(\frac{a}{W}\right)}f\left(\frac{a}{W}\right)\right)^2\right\}^{\phi} \tag{S29}$$

If the group of constants $\frac{D}{W}\left\{A\left(\frac{P}{BW}\right)^{n-1}\left(\frac{P}{BW}\sqrt{W}\right)^2\right\}^{\phi}$ is denoted as γ.

$$\frac{d(\frac{a}{W})}{dt} = \gamma \left\{ \left(\frac{1}{\left[g\left(\frac{a}{W}\right)\right]} \right)^{n-1} \left(\sqrt{\left(\frac{a}{W}\right)} f\left(\frac{a}{W}\right) \right)^2 \right\}^{\phi} \tag{S30}$$

This relationship can be normalised to the rate at a reference crack length a_0, to negate the sensitivity to the group of constants,

$$\frac{\frac{d\left(\frac{a}{W}\right)}{dt}}{\frac{d\left(\frac{a_0}{W}\right)}{dt}} = \frac{\left\{ \left(\frac{1}{\left[g\left(\frac{a}{W}\right)\right]} \right)^{n-1} \left(\sqrt{\left(\frac{a}{W}\right)} f\left(\frac{a}{W}\right) \right)^2 \right\}^{\phi}}{\left\{ \left(\frac{1}{\left[g\left(\frac{a_0}{W}\right)\right]} \right)^{n-1} \left(\sqrt{\left(\frac{a_0}{W}\right)} f\left(\frac{a_0}{W}\right) \right)^2 \right\}^{\phi}} \tag{S31}$$

It is then possible to plot normalised rate against normalised crack length. If m is assumed to be 5 and $a_0/W = 0.001$.

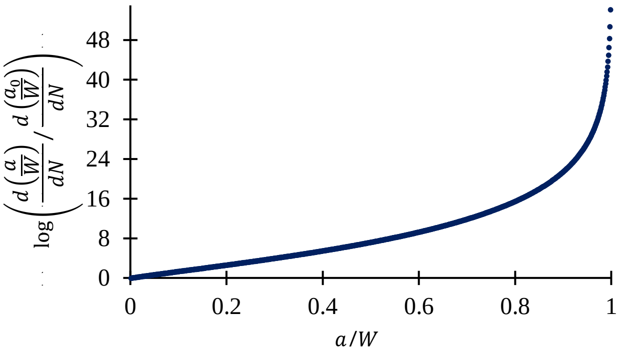

Figure S4: Normalised crack growth rate as a function of normalised crack length. Plotted from Equation (S31).

For compatibility with the Voight's postulation $\frac{d^2\Omega}{dt^2} = A\left(\frac{d\Omega}{dt}\right)^{\alpha}$ and the Failure Forecast Method fatigue crack growth must fulfil the requirement

$$\frac{df(\Omega)}{d\Omega} = Af(\Omega)^{\alpha-1} = A\left(\frac{d\Omega}{dt}\right)^{\alpha-1} \tag{S32}$$

If the damage state is determined by normalised crack length a/W then the relationship shown in Figure Figure S4 can be numerically differentiated to show good agreement with the form of equation equivalent to Equation (S32) as $f\left(\frac{a_0}{W}\right)$ is a constant and can be incorporated into the A parameter.

$$\frac{d\left[\frac{f\left(\frac{a}{W}\right)}{f\left(\frac{a_0}{W}\right)}\right]}{d\left(\frac{a}{W}\right)} = A\left[\frac{f\left(\frac{a}{W}\right)}{f\left(\frac{a_0}{W}\right)}\right]^{\alpha-1} \quad \text{(S33)}$$

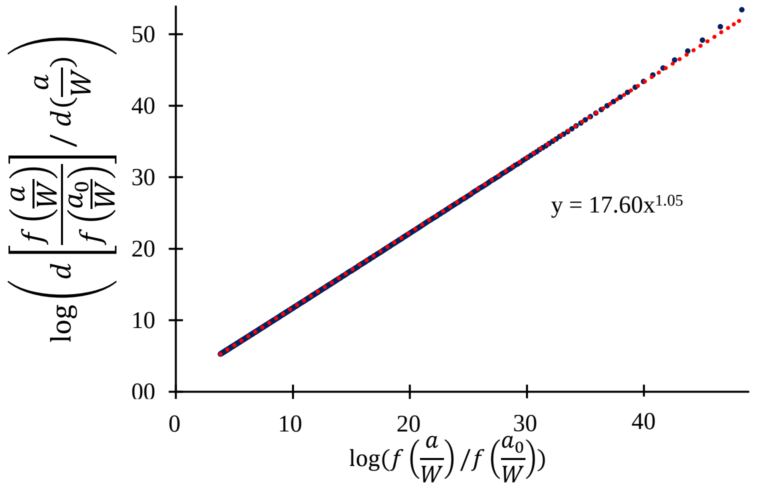

Figure S5: Normalised crack growth rate differentiated with respect to normalised crack length against normalised crack length. The power law relationship fulfils the criterion of Equation (6), indicating that it is consistent with Voight's postulated relationship, Equation (2).

The best fitting exponent depends on the chosen value for the parameter n, and subsequently, $\phi(n)$. Values for the best fitting exponent are given in Table S2.

Table S2: α values for a range of n. Values obtained by best fitting to Figure S5.

n	α
1	2.63
3	2.18
5	2.10
10	2.05
20	2.03

S4. Demonstration that the gravitational waves from inspiraling black holes behave according to Voight's postulated relationship

The separation between two inspiraling massive bodies will decrease at an increasing rate as they move closer together; additionally, the velocity of the bodies will increase. Both effects result in an increase in orbital frequency adhering to a known form; the gravitational waves that are emitted will be of twice the orbital frequency (*33*),

$$\frac{df}{dt} = \frac{\mathcal{M}^{\frac{5}{3}}}{\left(\frac{c^3}{G}\right)^{\frac{5}{3}} \frac{5}{96} \pi^{-\frac{8}{3}}} f^{\frac{11}{3}} \tag{S34}$$

where $\mathcal{M}$ is the 'chirp mass', a constant which is a function of the mass of the two bodies and c and G are the speed of light and the gravitational constant respectively. This characteristic behaviour is symptomatic of the underlying positive feedback and can be seen to be exactly analogous with Equation (5) and therefore conforms to Voights postulated equation, where α would be 19/11 for this case.

Supplementary Materials References

Listed in the main text.
